# Balancing acts: The posttranslational modification tightrope of flavivirus replication

**DOI:** 10.1371/journal.ppat.1012626

**Published:** 2024-10-28

**Authors:** RuthMabel Boytz, Maudry Laurent-Rolle

**Affiliations:** 1 Department of Microbial Pathogenesis, Yale University School of Medicine, New Haven, Connecticut, United States of America; 2 Section of Infectious Diseases, Department of Internal Medicine, Yale University School of Medicine, New Haven, Connecticut, United States of America; University of Iowa, UNITED STATES OF AMERICA

## Abstract

Posttranslational modifications (PTMs) such as phosphorylation, ubiquitination, SUMOylation, and ISGylation are involved in various cellular pathways, including innate immunity and disease processes. Many viruses have developed sophisticated mechanisms to modulate these host PTMs, either by inhibiting the interferon pathway or by enhancing the stability and function of viral proteins essential for replication. In this Pearl, we review the literature on how flaviviruses are impacted by and exploit posttranslational modifications to their advantage.

## Introduction

**Fig 1:**
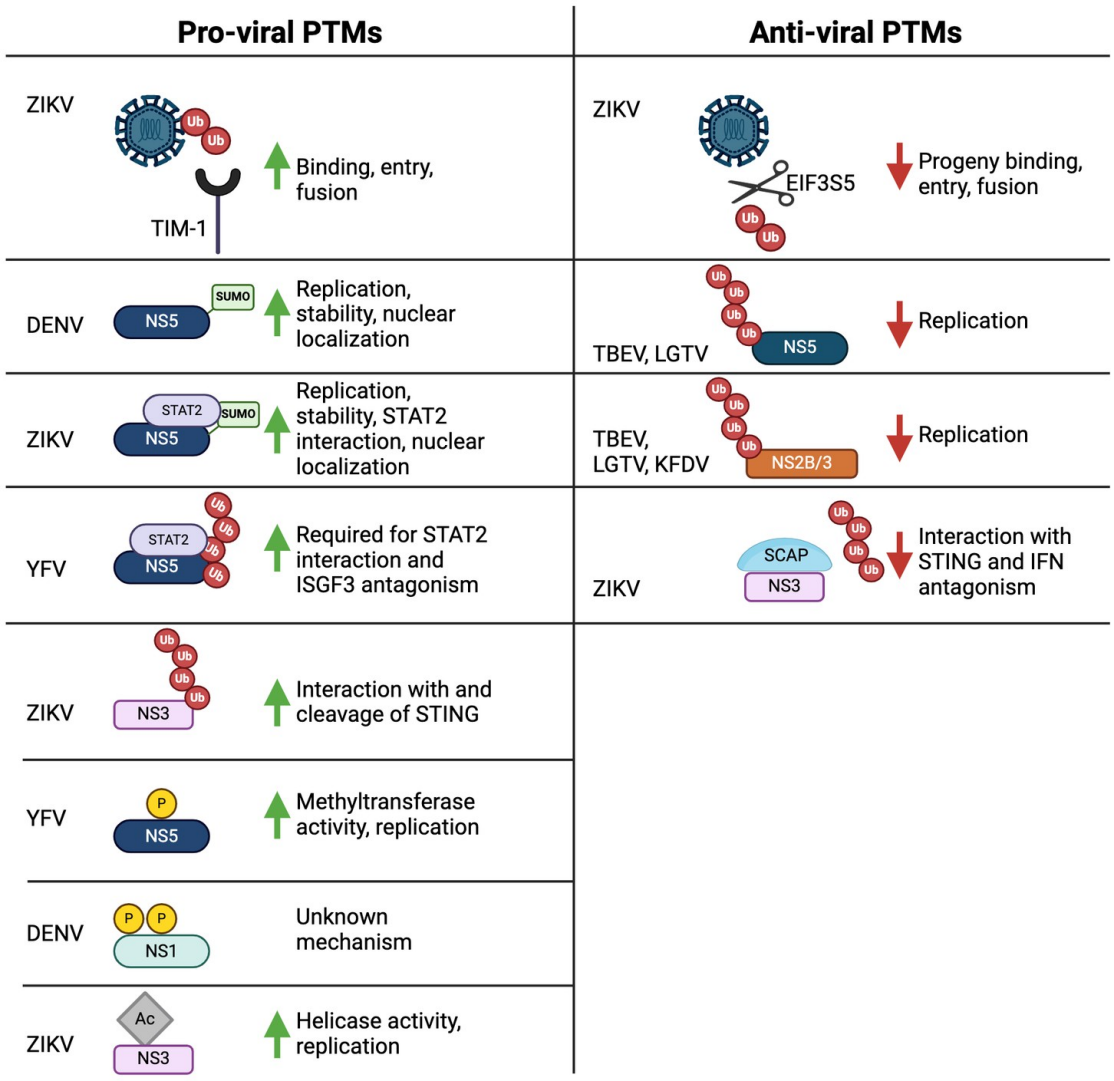
Flavivirus proteins undergo posttranslational modifications that can either enhance or suppress replication. ZIKV E is ubiquitinated by TRIM7 through poly-K63 linkages, which enhances ZIKV entry into target cells by increasing its interaction with the TIM-1 receptor. This effect is countered by the host deubiquitinase EIF3S5, which reduces the infectivity of ZIKV progeny virions. DENV NS5 is sumoylated, increasing its stability against proteasomal degradation, which promotes viral replication and NS5 nuclear localization. ZIKV NS5 is also sumoylated, enhancing its nuclear localization and interaction with STAT2, thus reducing ISG induction. YFV NS5 undergoes poly-ubiquitination via K63 linkages facilitated by TRIM23, a modification required for its IFN antagonism. Ubiquitinated NS5 binds STAT2, preventing the ISGF3 complex from associating with the ISRE of ISGs. Mitochondria release DNA (mtDNA) under infection-induced stress conditions. mtDNA is detected by STING, which activates IRF3 and IFN production. ZIKV NS3 interacts with and cleaves STING and favors the accumulation of degradative poly-K48 chains on STING. NS3 is ubiquitinated via poly-K27 linkages and this enhances its interaction with STING. This dampens RLR and cGAS-STING mediated production of IFN, creating conditions that favor viral replication. However, the host factor SCAP can bind NS3, preventing its ubiquitination and counteracting NS3’s STING antagonism. Phosphorylation of YFV NS5 promotes its methyltransferase activity and genome replication. DENV NS1 is phosphorylated at 5 potential sites by PKG, which is important for replication via an unknown mechanism. Reversible acetylation of ZIKV NS3 regulates its RNA binding and helicase activities. The host ubiquitin system can restrict viral replication by targeting viral proteins for ubiquitin-mediated degradation. The NS5 protein of TBEV and LGTV, as well as the NS2B/3 protein of TBEV, LGTV, and KFDV, are subjected to ubiquitination and subsequent degradation. Specifically, the ubiquitination and degradation of the NS5 protein from LGTV and TBEV are mediated by TRIM79α, while the ubiquitination and degradation of the NS2B/3 protein from TBEV, LGTV, and KFDV are mediated by TRIM5α. Fig 1 was made with Biorender.

Currently, over 3 billion people are at risk of infection by more than 70 flaviviruses [1]. Climate change and globalization are anticipated to expand this at-risk population and shift current disease distributions and transmission patterns [2]. Among these flaviviruses, dengue is particularly concerning due to its ability to cause serious illness and strain public health systems. Flaviviruses themselves encode a limited set of viral proteins. However, posttranslational modifications (PTMs), such as ubiquitin and ubiquitin-like modifications, significantly enhance the functional capabilities of these proteins. This enables flaviviruses to disrupt host proteins and processes, thereby enhancing their own replication and transmission. Host posttranslational modification systems on the other hand can also restrict viral replication. The most obvious being PTMs involved in antiviral interferon signaling. Therefore, PTMs can be viewed as a molecular tug-of-war between hosts and flaviviruses where the equilibrium can shift in favor of either the host or the virus. Here, we review the roles of PTMs to flavivirus proteins and how flaviviruses counteract host modifications (Fig 1). We note a significant research gap regarding the role of ubiquitin and ubiquitin-like modifications in mosquito vectors, particularly their impact on viral replication, transmission, host adaptation, and potential interactions with the RNA interference (RNAi) pathway. This topic will not be covered in this Pearl. Additionally, since the glycosylation of flavivirus proteins has been extensively reviewed elsewhere, we will focus specifically on ubiquitin and ubiquitin-like modifications.

### How do host cells use PTMs as an antiviral defense mechanism?

Interferon (IFN) and the production of interferon-stimulated genes (ISGs) are the first line of defense against viral infection and are essential for controlling viral replication. PTMs are critical for IFN production and signaling. Briefly, the cytosolic RNA sensors retinoic acid-inducible gene (RIG-I) and melanoma differentiation-associated gene 5 (MDA5) are ubiquitinated and, in the case of MDA5, ISGylated upon activation. Ubiquitination of their downstream signaling partners TNF receptor-associated factor 3/6 (TRAF3/6), NF-kappa B essential modulator (NEMO), and inhibitor of nuclear factor kappa (IkBa), and phosphorylation of the transcription factor interferon regulatory factor 3 (IRF3) leads to transcription of interferons (IFN) and activation of nuclear factor kappa B (NF-kB) [3].

Following IFN binding to receptors, the Janus kinases JAK1/2 and tyrosine kinase 2 (TYK2) are phosphorylated, which in turn phosphorylate signal transducer and activation of transcription 1/2 (STAT1/2), leading to their association with IRF9 and translocation of the interferon stimulated gene factor 3 (ISGF3) complex to the nucleus where ISGs are transcribed [4]. Together, IFN and ISGs establish a restrictive antiviral state.

Host cells directly modify viral proteins to restrict viral replication. For example, the E3 ubiquitin ligase TRIM79α promotes the lysosomal degradation of the viral RNA-dependent RNA- polymerase, nonstructural protein 5 (NS5) of Langat virus (LGTV) and tick-borne encephalitis virus (TBEV), thus reducing viral replication [5]. TRIM5α polyubiquitinates, via degradative K48-linkages, the NS2B/3 protein of LGTV, TBEV, and Kyasanur forest disease virus (KFDV) to induce their proteasomal degradation, which reduces viral genome replication [6]. In Zika virus (ZIKV) infection, LAMR1 recruits the deubiquitinase EIF3S5 to a complex with ZIKV E protein, allowing EIF3S5 to cleave both poly-K48 and poly-K63 ubiquitin chains from E, which reduces viral infection [7]. Furthermore, the NS3 protein of ZIKV is ubiquitinated by poly-K27 linkages, which promotes its ability to cleave and inactivate STING, thus reducing IFN production. However, binding by the host protein SCAP reduces this modification to inhibit NS2B/3-mediated STING cleavage and rescue IFN production and IFN-mediated restriction of viral replication [8]. This exemplifies the PTM tug-of-war between pro- and anti-viral PTMs and highlights the dynamic nature of these modifications (Table 1).

**Table 1:** Comprehensive table summarizing how different PTMs modulate flavivirus protein function.

Type of modification	Flavivirus	Process(es) affected	Reference number
Acetylation	ZIKV—NS3	Regulates switch from RNA binding to helicase activity, promotes replication	[35]
	WNV, DENV, YFV—NS3	Pro-viral	[35]
Glutathionylation	DENV, ZIKV—NS5	Reduces NS5 guanylyltransferase activity, possible effects on polymerase activity, possible effects on replication	[36]
Phosphorylation	YFV—NS5	Required for efficient NS5 methyltransferase activity, enhances genome replication	[32]
	DENV—NS5	Promotes replication	[33]
		May regulate nuclear localization	[31]
	DENV—NS1	5 potential phosporylation sites, 2 are required for producing infectious virions	[34]
Sumoylation	DENV—NS5	Increases NS5 stability, enhances NS5-mediated RIG-I degradation, promotes replication	[27]
	ZIKV—NS5	Nuclear NS5 localization, enhances NS5-STAT2 interaction and STAT2 degradation, promotes replication	[28]
Ubiquitination	ZIKV—E	Increases E—TIM-1 receptor interaction to enhance cell entry	[29]
	LGTV—NS2B/3	Proteasomal degradation, reduces genome replication	[6]
	TBEV—NS2B/3	Proteasomal degradation, reduces genome replication	[6]
	KFVD—NS2B/3	Proteasomal degradation, reduces genome replication	[6]
	ZIKV—NS2B/3	Enhances NS2B/3-mediated cleavage of STING, reduces IFN production	[8]
	LGTV—NS5	Lysosomal degradation, restricts replication	[5]
	TBEV—NS5	Restricts replication	[5]
	TBEV—NS4A	Enhances NS4A—STAT1 interaction, inhibits STAT1 phosphorylation	[23]
	YFV—NS5	Required for NS5—STAT2 binding, inhibits IFN production, enables viral replication	[26, 39]
	DENV—Capsid	Promotes p62 interaction and autophagic degradation of NS5, reduces replication	[41]

### Can flaviviruses alter the PTM landscape of host proteins?

Flaviviruses have evolved ways to antagonize IFN and ISG production, often by interfering with PTMs of signaling proteins. Several proteome-wide screens using Japanese encephalitis virus (JEV) [9], dengue virus (DENV) [10,11], and West Nile virus (WNV) [12] have demonstrated virus-induced alterations to host protein phosphorylation and ubiquitination landscapes. One of the best-studied examples is NS5-mediated antagonism of IFN signaling. DENV NS5 promotes STAT2 degradation by binding to STAT2 [13,14]. NS5 forms a complex with the E3 ubiquitin ligase UBR4 and STAT2, and this facilitates STAT2 degradation [15]. STAT2 ubiquitination and the role of UBR4 in this process have not been validated. However, it is likely that NS5 mediates the ubiquitination and proteasomal degradation of STAT2. Reduced STAT2 levels dampens ISG production creating a more favorable environment for DENV replication [15]. Similarly, the NS5 of TBEV, JEV, LGTV, ZIKV, Usutu virus (USUV), and WNV inhibit phosphorylation of STAT1, STAT2, JAK1 (TBEV), and TYK2 (TBEV, JEV), reducing ISGs [16–20]. In the case of ZIKV, NS5 association with STAT2 also leads to its proteasomal degradation, like DENV; however, in this case degradation is independent of UBR4 [19].

Other nonstructural proteins antagonize host PTMs. The NS2B/3 protein of ZIKV binds to stimulator of interferon response cGAMP interactor 1 (STING), a mitochondria-associated adaptor protein critical for IRF3 activation. This interaction promotes the accumulation of degradative poly-K48 modifications, leading to its proteasomal degradation and a reduction in IFN production [21]. The NS2A, NS2B, and NS2B/3 proteins of Kunjin virus (KUNV) can inhibit STAT2 phosphorylation and subsequent ISG production [22]. TBEV NS4A protein also interacts with STAT1 and STAT2 to inhibit phosphorylation. This impairs STAT1/2 dimerization, STAT1 homodimerization, and IRF9 binding and prevents ISGF3-mediated activation of ISGs [23]. WNV NS1 interacts with RIG-I and MDA5 to inhibit poly-K63 ubiquitination, thereby impairing their activation and resulting in the near-complete degradation of MDA5. However, it remains unclear whether this interaction occurs within the context of viral infection [24]. A final example again demonstrates the delicate balance of PTMs in favoring the host or the virus: ZIKV NS1 bridges caspase-1 and the deubiquitinase USP8, promoting USP8-mediated removal of poly-K11 ubiquitin chains from caspase-1 and preventing its proteasomal degradation. Caspase-1 activates NLRP3 inflammasome formation and cytokine IL-1β, which might be expected to be antiviral. However, stabilized caspase-1 also cleaves cGAS and this reduces IFN-I and ISG production, creating a more permissive environment for ZIKV replication [25]. Taken together, ZIKV uses a multifaceted strategy to dampen type I interferon production, thereby promoting its replication. Beyond IFN and ISG antagonism, the impact of flaviviral proteins on specific PTMs in autophagy proteins or other intrinsic pro- or antiviral pathways has not been extensively investigated, and this area warrants additional research.

### Do PTMs to flavivirus proteins themselves have pro-viral functions?

PTMs to viral proteins themselves can increase the protein’s functional capabilities. For example, ubiquitination of yellow fever virus (YFV) NS5 is required for YFV NS5-mediated antagonism of IFN signaling. The E3 ubiquitin ligase TRIM23 polyubiquitinates NS5 through K63-linkages at the K6 residue in NS5. This ubiquitination event is dependent on IFN signaling and is required for NS5-STAT2 binding and subsequent inhibition of ISG production [26]. In this example, ubiquitination of a viral protein by the host cell enables the viral protein to gain an IFN-antagonism function. This function is distinct from NS5’s role as the viral polymerase.

PTMs can directly promote viral replication. For instance, DENV NS5 protein is sumoylated by UBC9 which enhances NS5 stability against proteasomal degradation. NS5 sumoylation increases viral replication, likely through a combination of the increased polymerase activity and the antiviral antagonist activities of NS5 that results from increased NS5 stability [27]. A subsequent study in human brain endothelial cells also showed that sumoylation of DENV and ZIKV NS5, within a conserved SUMO-interacting motif (SIM) domain increased the stability of each NS5 protein and was required for NS5 nuclear localization [28]. Interestingly, despite the conserved SIM domain, ZIKV and DENV NS5 exhibit distinct sumoylation patterns. For ZIKV, disrupting NS5 sumoylation diminishes the NS5-STAT2 interaction and reduces its ability to suppress the induction of ISGs [28]. The SIM domain is conserved in YFV, JEV, WNV, and Powassan virus (POWV). Future studies may reveal flavivirus-specific and/or cell type-specific functions of NS5 sumoylation that underlie distinct tissue tropism and pathology associated with each virus.

PTMs can enhance viral entry into cells. The E protein of ZIKV is ubiquitinated via poly-K63 linkages by TRIM7 in producer host cells [29]. Ubiquitinated E is incorporated into viral progeny, and this ubiquitinated E has higher affinity for the proposed ZIKV receptor TIM-1. Importantly, the authors demonstrated that E ubiquitination occurs during infection of mouse models; can be detected in tissues; and affects viral titers. Subsequent studies support the role of ZIKV E ubiquitination in enhancing infection by showing that deubiquitination of E restricts replication [30]. This further demonstrates the tug-of-war between the host and the virus when it comes to the function of PTMs.

Besides ubiquitin and ubiquitin-like modifications, flavivirus nonstructural proteins have been reported to undergo phosphorylation [31–34], acetylation [35], and glutathionylation [36], although the effect of glutathionylation is unclear. YFV NS5 phosphorylation is critical for NS5 methyltransferase activity and genomic replication [32]. Protein kinase G (PKG) phosphorylates DENV NS5, and PKG activity enhances DENV replication, although the mechanism by which replication is enhanced remains to be determined [33]. DENV NS1 is also phosphorylated at 5 potential residues. Mutation of these sites differentially affects DENV replication, but again the function of each phosphorylation is unknown [34]. ZIKV NS3 undergoes dynamic, reversible acetylation by KAT5γ, and the switch between acetylated and non-acetylated forms appears to regulate viral RNA binding, dsRNA unwinding, and viral replication [35]. ZIKV clones with mutations to the acetylation site spontaneously revert to wild type after serial passage, underscoring the essential role of this modification in the replication cycle (Table 1).

### What are the practical implications of these host–virus PTM interactions?

The association between flavivirus envelope glycosylation and virulence/pathogenesis has been widely observed and studied [37]. Nonstructural protein modifications and ubiquitin or ubiquitin-like modifications have received relatively little attention but could have a significant impact on flavivirus virulence, pathogenesis, and host and tissue tropism. For example, in their research on ZIKV E ubiquitination, Giraldo and colleagues revealed a role for E ubiquitination in determining tissue tropism. Whereas E ubiquitination was important for ZIKV replication in the brain, testes, and uterus, it was dispensable in the spleen, liver, lungs, and muscles [29]. Guillain–Barre syndrome, sexual transmission, and microcephaly in infants born to infected mothers are features of ZIKV that might arise from infection of the brain, testes, and uterus, respectively [38]. ZIKV E ubiquitination might drive preferential infection of these organs over others and underlie characteristic symptoms and pathology associated with ZIKV infection.

PTMs may also serve as a species restriction factor for flaviviruses. For instance, mice are not competent hosts for DENV, YFV, or ZIKV unless they are deficient in IFN signaling. YFV NS5 must bind STAT2 to antagonize IFN signaling, and this interaction requires NS5 to be ubiquitinated [39]. YFV NS5 is not ubiquitinated in murine cells. Consequently, YFV NS5 cannot inhibit IFN signaling in murine cells, leading to the inability of YFV to establish a productive infection in immunocompetent mice [39]. Whether other proteins of flaviviruses exhibit similar species-dependent PTMs and their role in determining host competency remains an unexplored question. The implications extend beyond flaviviruses, particularly in predicting which animal viruses have the potential to infect and spread among humans.

An unresolved question in the field is whether the capsid protein is ubiquitinated and, if so, for what function. Byk and colleagues have demonstrated that ubiquitination in general is important for DENV genome uncoating, and that capsid is degraded by the proteasomal or lysosomal pathways, but did not demonstrate capsid ubiquitination [40]. A recent study found that DENV capsid appears to be ubiquitinated during viral infection, and that capsid interacts with an ubiquitin-associated domain (UBA) of the autophagy receptor p62, resulting in degradation of capsid and inhibition of replication [41]. Resolving this question will improve our understanding of essential host factors in the DENV replication cycle and may open novel avenues for therapeutic development.

Lastly, there are no approved antivirals for any flavivirus. Understanding the roles of PTMs and identifying commonly exploited host pathways could potentially facilitate the development of novel host-directed therapeutics that are broadly effective against multiple flaviviruses. Proteasome inhibitors such as bortezomib (Velcade) and carfilzomib are already in clinical use for the treatment of multiple myeloma. Notably, bortezomib has been reported to inhibit ZIKV and DENV replication by interfering with viral polyprotein cleavage through the endoplasmic reticulum-associated degradation (ERAD) pathway [42]. Host-targeted therapies offer the advantage of circumventing viral resistance mechanisms and could decrease the public health burden of multiple flaviviruses without the need to develop a unique drug for each virus.
